# Simultaneous Intrusion and Retraction of Maxillary Anterior Teeth in Class II Malocclusion Using the Kalra Simultaneous Intrusion and Retraction (K-SIR) Loop: A Case Report

**DOI:** 10.7759/cureus.53241

**Published:** 2024-01-30

**Authors:** Shefali Singh, Rizwan Gilani, Shruti Rathi

**Affiliations:** 1 Orthodontics and Dentofacial Orthopaedics, Sharad Pawar Dental College and Hospital, Datta Meghe Institute of Higher Education and Research, Wardha, IND

**Keywords:** space closure, loops, retraction, intrusion, k-sir loop

## Abstract

The straight-wire device offers the best finishing potential and control. This case study focuses on the treatment of severe deep bite and Class II malocclusion involving first premolar extraction of the upper arch using a Kalra Simultaneous Intrusion and Retraction loop. Using minimal force and creating enough space for anterior teeth to retract while maintaining the Class II molar relationship was the aim of the therapy. Due to the unsightly excessive maxillary incisor showing at rest, the decision was made to intrude anterior teeth to treat a deep overbite. Good and consistent changes occurred post-treatment.

## Introduction

Individuals who exhibit crowding and/or protrusion frequently undergo extraction procedures, necessitating a deep comprehension of biomechanics. Sliding and loop mechanics are the two fundamental subtypes of space closure mechanics. In sliding mechanics, the wire and bracket location play a significant role in tooth movement; nevertheless, the binding between the bracket and archwire negates the simplicity of friction mechanics and may lead to unfavorable side effects, including deep bite and uncontrollably tipping teeth [1].

In orthodontics, loop mechanics refer to the use of wire loops or bends in orthodontic wires to achieve specific tooth movements. These loops can be designed to apply forces that contribute to tasks such as rotating, tipping, or aligning teeth. Different types of loops, such as omega loops, T-loops, Kalra Simultaneous Intrusion and Retraction (K-SIR) loops, or closing loops, are utilized based on the treatment plan and desired tooth movement [2].

The design and placement of these loops play a crucial role in the overall effectiveness of the treatment [3]. They provide a means for controlled force application, allowing orthodontists to address various issues in tooth alignment and bite correction. Correcting deep bite is one of the primary objectives of orthodontic therapy, and it is the most prevalent malocclusion and one of the hardest to effectively cure. Thus, a correct diagnosis, a meticulous treatment plan, and an effective appliance design are necessary for the best possible care of deep bite [4].

The segmented loop mechanics of Burstone and Nanda reported in 1962 have been modified to create the K-SIR archwire. It consists of a continuous 0.019” × 0.025” TMA archwire with closed U loops measuring 7 mm × 2 mm at the extraction sites [5].

## Case presentation

An 18-year-old female patient reported to the Department of Orthodontics 18 months back with a chief complaint of upper front teeth being forwardly placed.

**Figure 1:**
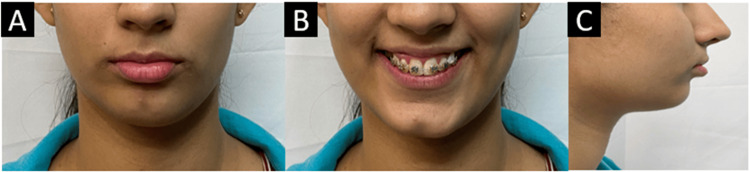
Pretreatment extraoral photographs. (A) Frontal. (B) Smiling. (C) Profile.

Extraoral examination revealed a symmetrical mesoprosopic face form. The patient had a convex profile and a retrusive chin on profile examination. Lips were competent, the nasolabial angle was acute, and the mentolabial sulcus was deep (Figure 1).

**Figure 2:**
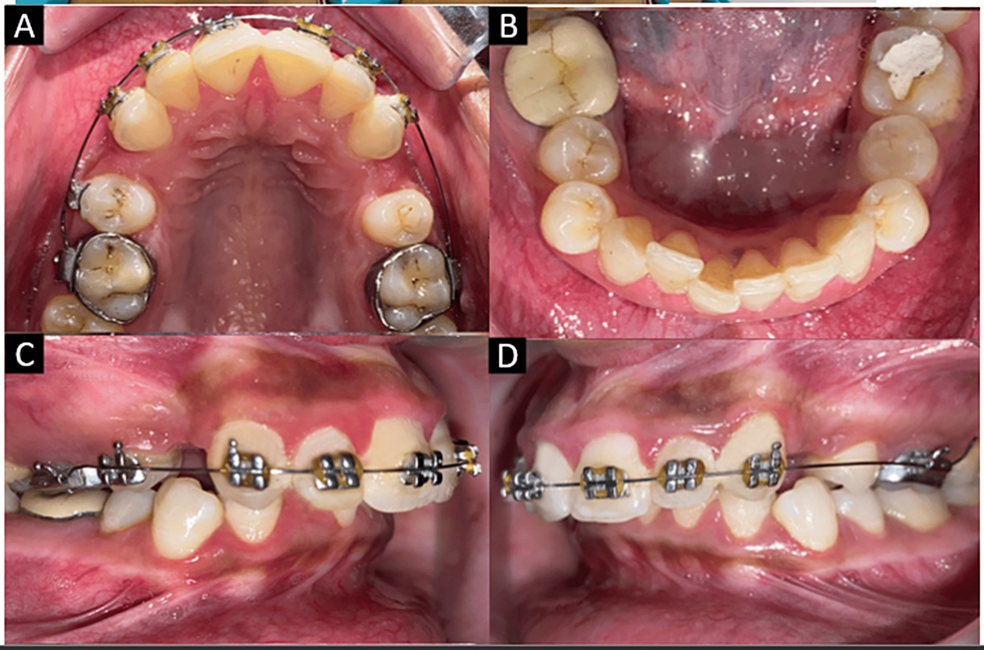
Pretreatment intraoral photographs. (A) Maxillary arch. (B) Mandibular arch. (C) Right lateral. (D) Left lateral.

Intraoral examination revealed all permanent teeth erupted except the third molars. Extraction of upper premolars had been done along with strap-up of the upper arch in a private clinic. The lower arch was crowded and the upper anterior teeth were proclined. The anterior deep bite was seen with complete overlapping of the incisors. The upper and lower arches were U-shaped with Class II molar and canine relationships on both the right and left sides (Figure 2).

**Figure 3:**
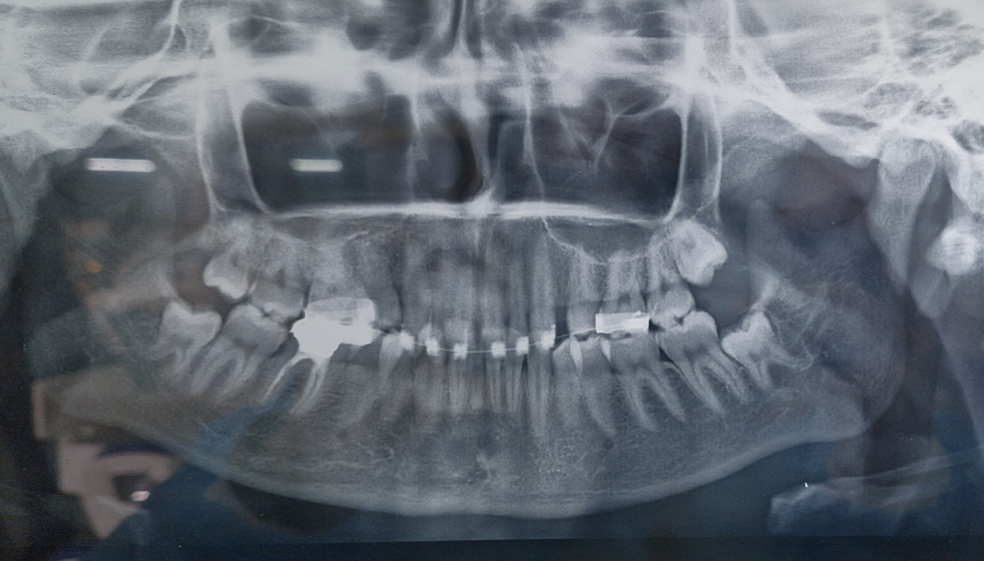
Pretreatment orthopantomogram.

Pretreatment orthopantomogram (OPG) revealed all teeth present in all four quadrants including all third molars (Figure 3).

**Figure 4:**
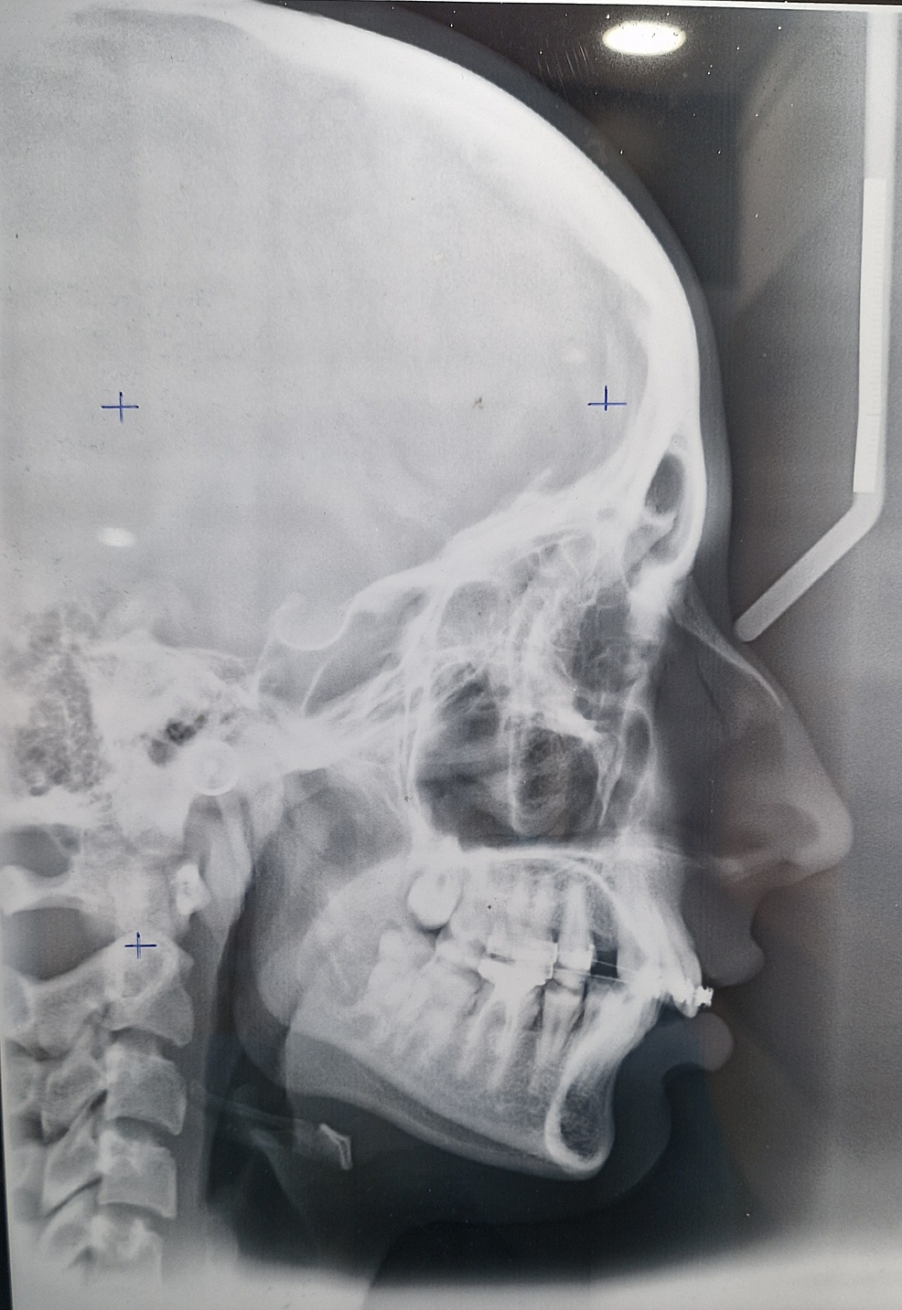
Pretreatment lateral cephalogram.

Cephalometric analysis revealed that the patient was in cervical vertebrae maturation index stage IV (completion) and had Class II skeletal bases, horizontal growth pattern, and proclined upper incisors depicted by 1 to NA angle of 26 degrees (Figure 4).

Model analysis revealed proclination of the maxillary arch by 4 mm and the mandibular arch by 2 mm. Linder Harth index revealed a narrow maxillary arch, and Bolton’s ratio for the anterior region and overall were 66% and 85%, respectively. Space discrepancy in the maxillary arch was 8 mm and for the mandibular arch was 2.5 mm.

Treatment objectives were to align upper and lower arches with correction of lower crowding and anterior deep bite, reduce proclination of the upper anterior teeth (1 to NA angle being 26 degrees), achieve normal overjet and overbite, improve the profile, maintain Class II molar relationship, and achieve Class I canine relationship on both sides.

Orthodontic phase

**Figure 5:**
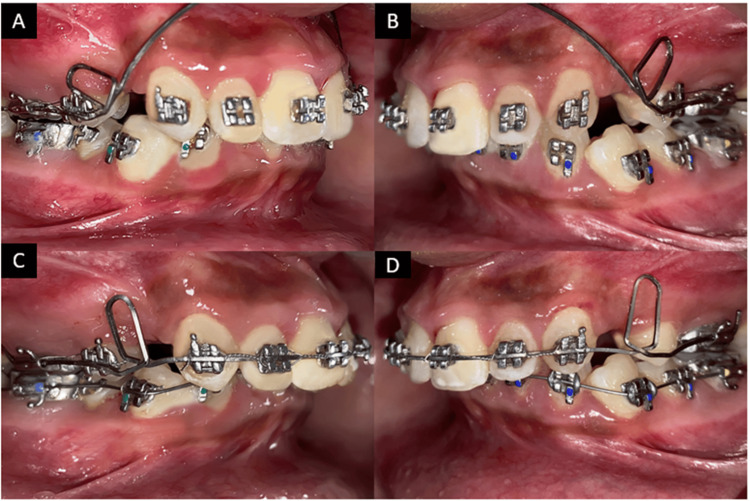
Intraoral photographs with the appliance. (A and B) Activated K-SIR loop. (C and D) Ligated K-SIR loop. K-SIR: Kalra Simultaneous Intrusion and Retraction

As the upper first premolars were already extracted prior in an outside clinic with strap-up using an MBT 0.022 slot of the upper arch, treatment was started by strap-up of the lower arch using the same MBT prescription. Initial leveling and alignment of the arches were started with round wires followed by rectangular wire until 0.019 × 0.025 stainless steel wire. At the extraction sites, there were closed 7 mm × 2 mm U-loops on a continuous 0.019” × 0.025” TMA archwire without any curve of Spee (Figure 5). The loops were activated extraorally, and when the archwire was placed in the molar tubes, the archwire rested gingivally in the vestibule. This wire was pulled and ligated in the bracket slots.

After relieving the crowding in the lower arch and leveling and alignment of the upper arch, retraction of the upper arch was started using the K-SIR loop to achieve intrusion and retraction at the same time.

Results

**Figure 6:**
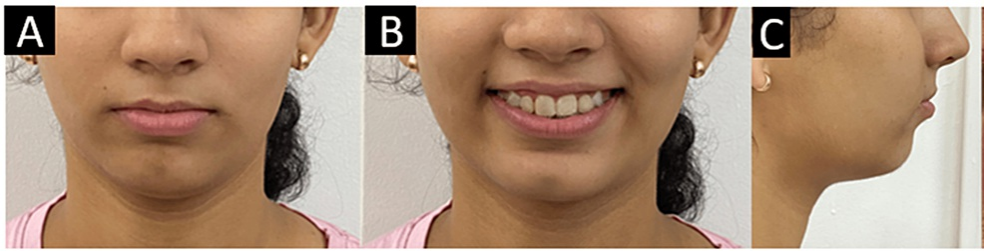
Post-treatment extraoral photographs. (A) Frontal. (B) Smiling. (C) Profile.

After 18 months of fixed orthodontic treatment with the K-SIR loop, retraction of the anterior teeth was achieved along with a significant amount of intrusion causing correction of deep bite and proclination. Crowding in the lower arch was relieved. The patient profile improved extraorally (Figure 6).

**Figure 7:**
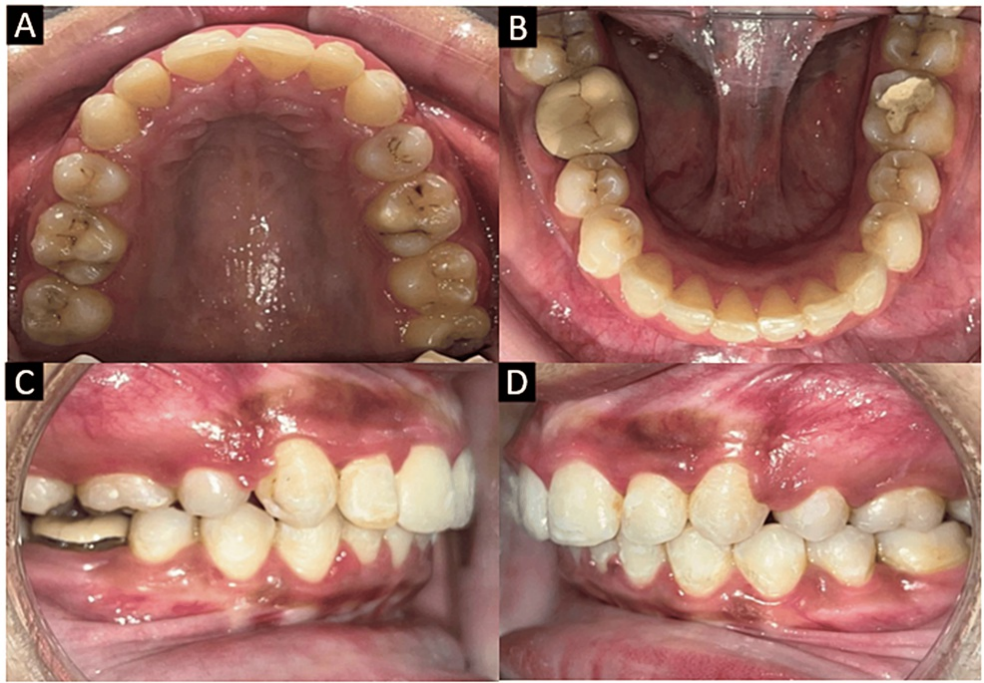
Post-treatment intraoral photographs. (A) Maxillary arch. (B) Mandibular arch. (C) Right occlusion. (D) Left occlusion.

Space closure in the upper arch and crowding were relieved in the lower arch while maintaining Class II molar relation intraorally (Figure 7).

**Figure 8:**
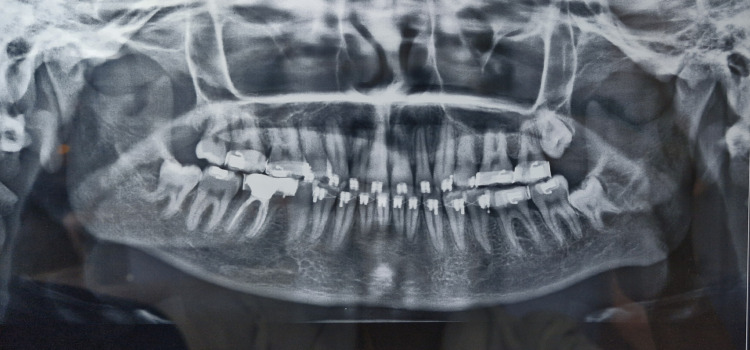
Post-treatment orthopantomogram.

Post-treatment OPG was taken just before debonding to ensure root parallelism and the radiograph revealed parallel roots of all teeth (Figure 8).

**Figure 9:**
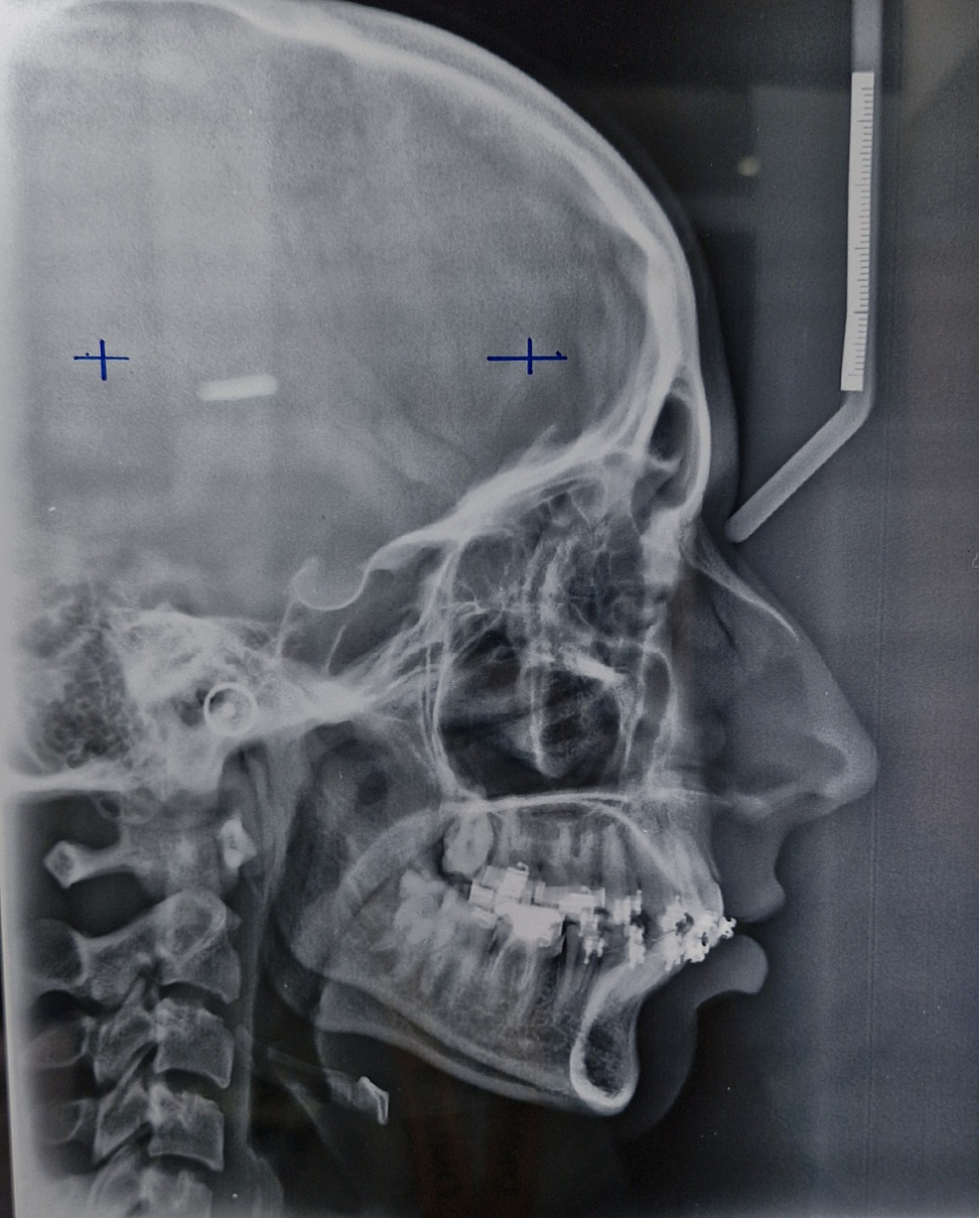
Post-treatment lateral cephalogram.

Cephalometric analysis of the post-treatment lateral cephalogram revealed a significant reduction in 1 to NA angle from 26 degrees to 22 degrees which indicated a reduction in the proclination of the upper anterior teeth (Figure 9).

## Discussion

With the edgewise technique, anterior teeth retraction typically occurs in two separate stages, namely, incisor retraction and canine retraction. On the other hand, the incisors and canines are retracted together in the Begg and Tip-Edge procedures. Because molar anchoring is conserved, separate retraction is justified in the edgewise approach [6]. However, Burstone and Nanda have shown molar anchoring control, with en masse retraction of the anterior teeth achieved by non-frictional loop mechanics that compares favorably to traditional edgewise sliding mechanics. Burstone and Nanda introduced the segmented loop mechanics which was modified into the K-SIR archwire. At the extraction sites, there are closed 7 mm × 2 mm U-loops on a continuous 0.019” × 0.025” TMA archwire. A V-bend of 90 degrees at the level of each U-loop allows for natural movement of the teeth and prevents them from tipping into the extraction gaps. On the first molar, a 60-degree V-bend that is posterior to the interbracket distance center causes a greater clockwise moment [5]. An off-center V-bend increases the moment on the molar, which increases the molar anchoring and anterior tooth intrusion. An anti-rotation bend of 20 degrees is inserted into the archwire immediately distal to each U-loop to stop the buccal segments from rolling mesiolingually [7]. The second premolars are eliminated to increase the interbracket space between the attachment. This enables the physician to apply the off-center V-bend’s mechanics. Until the entire extraction space has been closed, the reactivation of the archwire should be done every six to eight weeks [8].

With different degrees of overbites, the archwire is adjusted to cause the closure of extraction areas in minimum and moderate anchorage scenarios. The 0.019” × 0.025” TMA has enough stiffness to produce the necessary moments and enough strength to withstand deformation [9]. Simultaneously, the archwire’s design and TMA’s material qualities result in comparatively fewer forces, a lower range of activation, and a lower rate of load deflection, which permits the closure of the space within eight weeks. TMA may be activated twice as much as stainless steel without permanently deforming, and it produces half the force per unit activation. When compared to traditional edgewise mechanics, the K-SIR archwire shortens treatment time because the retraction of the canines and incisors occurs simultaneously with anterior six teeth intrusion. Furthermore, the six anterior teeth are retracted collectively, which prevents the ugly area distal to the incisors from developing if the canine retraction is done individually [10].

## Conclusions

The K-SIR archwire is primarily used to retract front teeth in patients who require maximum molar anchorage in addition to front tooth intrusion following first premolar extractions and who have severe overjet and deep overbite.

Although there are several advantages of using the K-SIR loop such as retraction as well as the simultaneous intrusion of teeth in cases of deep bite and lack of friction between the bracket and archwire during space closure, it has several disadvantages as well. These disadvantages include a lack of patient compliance, the range of activation is quite constrained, and it produces a moment-to-force ratio that is far from optimal if controlled tipping or translation is desired. An alternate treatment for this case can be a retraction of the anterior for the closure of extraction space using friction mechanics.
